# Barriers to and Facilitators of Hepatitis B Vaccination among the Adult Population in Indonesia: A Mixed Methods Study

**DOI:** 10.3390/vaccines11020398

**Published:** 2023-02-09

**Authors:** Putri Bungsu Machmud, Amand Führer, Cornelia Gottschick, Rafael Mikolajczyk

**Affiliations:** 1 Institute of Medical Epidemiology, Biometrics, and Informatics (IMEBI), Interdisciplinary Center for Health Sciences, Medical School of the Martin Luther University Halle-Wittenberg, Magdeburger Str. 8, 06112 Halle (Saale), Germany; 2 Department of Epidemiology, Public Health Faculty, Universitas Indonesia, Jl. Prof Dr Bahder Djohan, Depok 16424, Jawa Barat, Indonesia

**Keywords:** adult, hepatitis B, Indonesia, mixed methods, vaccination

## Abstract

To reach the goals of the Global Hepatitis Elimination 2030 program, Indonesia is now preparing a new regulation for hepatitis B vaccinations for adult population. This study aimed to determine the factors influencing vaccine uptake for hepatitis B in the adult population, and identify barriers to, and facilitators of, hepatitis B vaccination programmes. An explanatory sequential mixed methods design was implemented in this study. We conducted a survey involving 893 participants in the general population followed by 14 in-depth interviews with health providers. The survey found that only 15% (95% confidence interval 13–18%) of participants received at least one dose of the hepatitis B vaccine. Factors associated with vaccine uptake were, living in Yogyakarta compared to living in Aceh, having secondary and higher education compared to primary education, working as a health worker compared to working in other sectors, and having health insurance that covered hepatitis B vaccination compared to not having such health insurance. Our qualitative study also identified several barriers to the adult hepatitis B vaccination programme in Indonesia such as the high cost of vaccination, lack of vaccine availability in certain areas, limited human resources to implement the hepatitis B vaccination programme, and the ineffective dissemination of hepatitis B vaccination. This study highlights that accessibility and affordability of vaccinations are important determinants of vaccination uptake that should be taken into account when planning vaccination campaigns.

## 1. Introduction

The World Health Organization states that, in the mission to eliminate hepatitis viruses as a global threat, all countries should aim to reduce hepatitis incidence by 90%, and mortality rates by 65%, compared to the basis data of 2015 [1]. This target is to be achieved through several programmes, one of which is called “Leaving No One Behind”. The goal of this programme is to provide access to prevention services (vaccination and testing) and treatment of hepatitis to everyone, including drug users, people in prisons, migrants, and health care workers, which are high-risk populations for hepatitis [2].

Indonesia is one of 11 countries that together carry almost 50% of the global burden of chronic hepatitis, and is rated as an intermediate-to-high hepatitis B virus endemic region [3]. The 2013 Indonesian National Health Survey reported that the percentage of hepatitis infection reached 7.1%, with the most common type of hepatitis virus being hepatitis B (21.8%) [4,5]. The highest incidence of all hepatitis (reported based on diagnoses and symptoms) was in the age group of those 45 to 54 years (1.4%), living in rural areas (1.4%), and people working as farmers/fishers/labourers (2.0%) [4,5]. 

The country is working to develop a strategic roadmap to gradually eliminate viral hepatitis [6]. The strategy started with a programme that reinforced issuing comprehensive hepatitis virus regulations through health promotion, prevention, early detection, and sub-management approaches in 2015 [7], followed by a comprehensive vaccination schedule for children in 2017 that also includes hepatitis B vaccination (a zero dose of hepatitis B vaccine, followed by three additional doses) [8]. In addition, in the same year, Indonesia’s Ministry of Health launched a programme that focuses on the elimination of vertical transmission from mother to child [9]. Furthermore, to meet the goals of the Global Hepatitis Elimination 2030 programme [10], Indonesia is currently preparing a new regulation for hepatitis B vaccinations for adults, which focuses on high-risk populations and aims to gradually offer voluntary vaccinations to health care workers, starting in 2022 [6].

Despite all these activities, no studies have yet assessed the factors that affect vaccine uptake for hepatitis B among the adult population in Indonesia. Some studies from other developing countries have investigated how demographic factors, such as age, sex, education, ethnic group, residency, marital status, monthly income, health insurance, and occupation, are associated with vaccine uptake for hepatitis B [11,12,13,14,15,16]. In addition, some studies have also found that lifestyle factors [17,18,19] are also associated with being vaccinated for hepatitis B. However, most of these studies analysed the influences on vaccination status from the receiver side only—that is, the population. 

We argue that a population’s vaccination status is not influenced by acceptance from the vaccine’s receivers alone, but that health care providers, and their ability to deliver accessible and acceptable services, are crucial as well [20]. In addition, there is so far very little research looking at those determinants of vaccination uptake in the Indonesian context. Therefore, we aimed to determine factors influencing vaccine uptake for hepatitis B among the adult population and identify barriers to, and facilitators of, hepatitis B vaccination programmes in two groups, namely those to be vaccinated and vaccination providers, to achieve a deeper understanding of the factors related to vaccination. 

## 2. Materials and Methods

An explanatory sequential mixed methods study was conducted [21]. The quantitative part consisted of a survey that was conducted in 2020 in two provinces in Indonesia: Aceh and Yogyakarta. These provinces were selected to achieve a maximal contrast: Yogyakarta Province had the highest vaccination coverage within the existing programme (83.7% with complete vaccination, 16.3% with incomplete vaccination, and none without vaccination), while Aceh Province was reported to have the lowest coverage (19.5% with complete vaccination, 16.3% with incomplete vaccination, and 40.9% never vaccinated), based on the Indonesia National Health Survey 2018 [5]. In addition, both provinces differ with respect to average income and education, and have very different socio-religious imprints [4].

### 2.1. Quantitative Study

The survey aimed to investigate the knowledge, risk-perception, willingness to get vaccinated, and vaccination status, as well as the reasons for not getting vaccinated in relation to hepatitis B, among the adult population. The data were collected through face-to-face interviews conducted by the interviewers. We involved two local enumerators, who were trained and supervised by the field coordinator. Details on the methodological approach for the quantitative part have been published elsewhere [22]. In brief, an institution-based cross-sectional survey was conducted from February to March 2020 in Aceh and Yogyakarta. We included staff that worked in a health centre with both medical and non-medical backgrounds, and outpatients who registered at the health centre on the same day that the data were collected.

Staff (N = 508) and outpatients (N = 492) in sixteen selected health centres were randomly sampled from employment data and the patient register, respectively. Participants who reported in the study questionnaire that they did not get vaccinated because they have already been infected with hepatitis B were retrospectively excluded from the analysis. Participants were asked to respond to the question ‘Have you ever received an adult hepatitis B vaccination?’ The possible response was dichotomous: yes or no. 

Sociodemographic variables included in the study were age group, sex, marital status, religion, residency, education, occupational status, and profession within the health centre, monthly income, and whether the participant had health insurance covering hepatitis B vaccination costs. Furthermore, knowledge about hepatitis B infection and vaccination, risk perceptions, exposure to information regarding hepatitis B, and reasons for not getting vaccinated against hepatitis B were also included in this study. For more details on the questionnaire, see the Tables S1–S3. 

The continuous variables were presented as means with their 95% confidence interval (95% CI), while the categorical variables were reported as absolute and relative frequencies. Logistic regression was used to assess the association between the outcome variable (vaccine uptake) and the independent variables (sociodemographics, knowledge, and risk perception regarding hepatitis B infection). First, all independent variables that had an association with the outcome variable based on previous studies [12,15,23,24,25,26,27,28] were included into a univariable logistic regression model, and crude odds ratios (COR) were obtained (model 1, Table 1). Subsequently, a multivariable logistic regression was applied to calculate mutually adjusted odds ratios (AOR) (model 2, Table 1). In the multivariable model only sociodemographic variables were included. Attitudinal variables were not included, as they are likely in the causal chain for vaccination uptake [29]. We also did not include the variable of monthly income in the multivariable model, due to too many missing responses (>80%). All analyses were performed using the Statistical Package for the Social Sciences software (SPSS version 20, Chicago, IL, USA) and STATA 16.1.

### 2.2. Qualitative Study

The qualitative study assessed the current hepatitis B programme as well as barriers to, and facilitators of, hepatitis B vaccination from the government’s perspective, through in-depth interviews. We recruited informants from the local health offices on a city/district level from the same areas as covered in the quantitative study. In these health offices, we targeted employees who were responsible for programmes related to hepatitis B, vaccination, and health promotion. In addition, we recruited informants from the Ministry of Health’s three directorates: the Directorate of Communicable Disease Prevention and Control, the Directorate of Immunization, and the Directorate of Health Promotion and Community Empowerment. In total, 49 informants from the Ministry of Health, and four health offices were actively involved with programmes related to this study. 

Prior to the interviews, heads of departments received information material introducing the study and signed a memorandum of understanding that they would allow their employees to participate in the study. They then delegated one of their employees to participate in the study, whereby they were asked to choose somebody actively involved as a programme implementer. 

In the subsequent step, we sent an invitation letter to the selected informants by an e-mail containing an introductory letter introducing the research topics, detailed research information, and an informed consent form. The interviews were conducted from May 2021 to July 2021. The interviews were conducted in Indonesian (Bahasa) by the first author (PBM), who is Indonesian. Each interview took between 45 and 90 min. 

The interviews were video recorded and transcribed verbatim in Bahasa by native speaking research assistants. Then, transcripts were coded by the first author using deductive thematic analysis; that is, the data were categorised based on the ‘high-quality health system framework foundations’ [30]. Hereby, the researcher made notes on the label codes, which were then classified into themes, based on their five foundations of a high-quality health care system, which is part of the revolution in area of the sustainable development goals (SDGs) that has appeared in the literature recently. The foundations consist of population, governance and financing, platform, workforce, and tools [30].

Population is defined as a group of people who receive the benefits of the health system. In addition, population also works as an important partner that improves their own health outcomes and the present health care system [30]. In this study, population consists of the people in the communities being studied, including their health needs, knowledge, health literacy, preferences, and cultural norms. 

Strong governance and financing are needed in a high-quality health care system to provide regulation, organise care, and institutionalise accountability to the citizens [30]. The component of governance includes leadership (political commitment, change management, and policies), financing (funding, insurance and purchasing, and payment), learning and improvement (institutions for evaluation, measurement, and improvement, learning communities, and trustworthy data), and intersectoral factors (roads, transport, water and sanitation, the electric grid, and higher education) [30]. 

The third foundation of a high-quality health care system in the era of the SDGs is platform. In this study, a component of platform related to hepatitis B vaccination coverage in Indonesia is the assets that health facilities have, including the number and distribution of health facilities and geographic access to health facilities that provide hepatitis B vaccination [30]. 

Hepatitis B vaccination coverage in Indonesia also depends on the health workforce. We included an adequate number and distribution of health care workers, the skill and professionalism of health care workers, and the motivation of health care workers, as components of the workforce in this study [30]. Finally, we also included tools, defined as equipment and information systems related to promotion of the hepatitis B programme [30]. All qualitative analyses were performed using MAXQDA Analysis Pro 2020 (version 20.4.2).

### 2.3. Data Integration

We integrated the findings from the quantitative and the qualitative arm using a matrix joint display technique, which was adapted from the pillar integration process (PIP) concept by combining the findings of the survey and the in-depth interviews [31]. The PIP started by listing all the findings from both the survey and in-depth interviews, followed by a process matching the findings between the population side and government side. 

## 3. Results

### 3.1. Quantitative Findings

#### 3.1.1. Respondents’ Characteristics

In total, 1000 participants were approached, of these, 98 refused to participate from the beginning and seven dropped out during the interview. Two participants who had previously been infected with hepatitis B were excluded retrospectively from the analyses. The participants’ median age was 35 (interquartile range (IQR) 26–42) years. Most of the participants were women (n = 726; 81.3%), and the majority (n = 407, 56.8%) were health care workers. For more details on the demographic characteristics of the participants, see Table 2.

#### 3.1.2. Knowledge, Risk Perception, and Vaccination Status among Respondents

Table 3 describes the respondents’ knowledge and risk perception about hepatitis B infection, as well as their vaccination status. The mean knowledge scores concerning hepatitis B infection and vaccination among health centre staff who worked in high-risk and low-risk units (20.36; 95% confidence intervals (CI): 19.92–20.80 and 18.40; 95% CI: 17.73–19.07, respectively) were higher than the knowledge scores of non health care workers (9.08; 95% CI: 8.46–9.72). Similarly, the mean risk perception scores regarding hepatitis B differed between health centre staff (13.44; 95% CI: 13.26–13.62 in high-risk unit and 13.00; 95% CI: 12.72–13.28 in low-risk units) and non health care workers (11.65; 95% CI: 11.41–11.89). About one sixth (n = 136 of 893, 15.2%) of the participants were vaccinated with at least one dose of the hepatitis B vaccination.

#### 3.1.3. Information Exposure and Reason Not to Be Vaccinated among Respondents

Among the participants who had received hepatitis B information (n = 729, 81.6%), most of them (n = 527, 72.3%) had received information from a health provider; the second source of information was the media (n = 397, 54.5%). Among the participants who had received hepatitis B information from the media, 63.2% (n = 251) and 54.2% (n = 215) reported television and social media as platforms to obtain that information, respectively (Table 4). 

**Figure 1:**
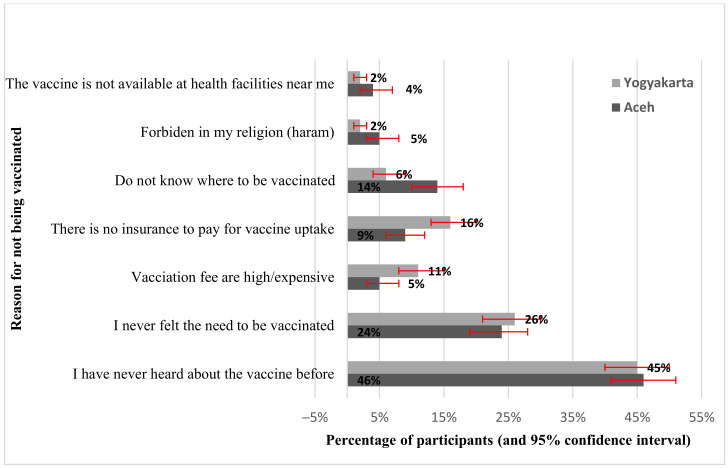
Reasons not to be vaccinated.

The main reasons for not being vaccinated were that respondents had ‘never heard about hepatitis B vaccination for an adult before’ and ‘never felt the need for vaccination (for hepatitis B for an adult)’, reported by 45.4% (n = 344) and 24.7% (n = 187) of the unvaccinated participants, respectively. Further reasons for not getting hepatitis B vaccination are illustrated in Figure 1.

#### 3.1.4. Variables Associated with Vaccine Uptake

The multivariable analyses variables that were statistically associated with vaccine uptake involved all respondents (vaccinated or never). These four factors were found to have a statistically significant association with vaccine uptake in the adult population (Table 1): living in Yogyakarta compared to living in Aceh, having secondary or higher education compared to primary education, working as a health care worker in a low-risk unit or in a high-risk unit compared to being a non-health care worker, and having health insurance that covered hepatitis B vaccination compared to not having such health insurance.

### 3.2. Qualitative Findings

In total, 14 informants were included in this study. The mean age of the informants was 44.7 (standard deviation = 6.21) years. The following text, and Table 5, describe the qualitative findings, categorised according to the foundations of the high-quality health care system framework. 

#### 3.2.1. Population

The informants’ narratives highlighted that knowledge about hepatitis B vaccination, and cultural norms in the population, act as important factors for adult uptake of hepatitis B vaccination. For instance, informants said that one of the common barriers to vaccination programmes is a lack of knowledge concerning the benefits of vaccination, which results in vaccine hesitancy among particular populations in Indonesia. Furthermore, cultural norms, such as the tradition of *manut* (meaning ‘obey the command’ in Javanese), were also mentioned by informants in the interviews as influencing vaccination uptake among particular populations. For instance, an informant from Yogyakarta and Gunungkidul said that it was not difficult to ask Yogyakarta’s people to vaccinate once the king ordered the programme—this statement being related to the culture of Yogyakarta, which is famous for having a population that complies with its king (who is currently also in the role of governor). Thus, it is unsurprising that Yogyakarta achieved almost complete vaccination coverage in recent years. In contrast, informants argued that the suspicion that the vaccine was rumoured to contain pork (the ‘halal controversy’) was a common reason for the population in Aceh to reject vaccination (Aceh is an Indonesian region where Sharia law is implemented).

#### 3.2.2. Governance and Financing

We found three barriers to hepatitis B vaccination programmes that related to governance and financing in Indonesia. Firstly, some informants indicated that the hepatitis B programme, including hepatitis B vaccination, is ‘more unpopular’ compared to the other national priority programmes: health insurance, maternal and child health, stunting reduction, tuberculosis, COVID-19, and the healthy living community movement (GERMAS in Indonesian). In fact, one informant confessed that he did not even know that hepatitis B vaccination was available. Secondly, informants believed that the cost of hepatitis B vaccination is one of the common reasons not to get vaccinated, both for health care workers and the general population, as they have to pay for the vaccination themselves. Finally, informants argued that another barrier to the hepatitis B programme is the lack of data related to hepatitis B. All the informants who were part of the hepatitis B programme said that without accurate data regarding hepatitis B cases, treatment, and adult vaccination in Indonesia, it is difficult for health programmers to analyse hepatitis B-related problems, including assessing the urgency of adult vaccination.

#### 3.2.3. Platforms

In this study, some informants believed that the low availability of the vaccine poses a barrier to vaccination: In some areas in Indonesia, people need to go to urban areas to obtain it. This issue is not restricted to hepatitis B programmes: limited health facilities and geographic access to facilities also impacts the coverage of other vaccination programmes—for example, for children. In this context, one informant, who handles the national vaccination programme, reported that timely birth-dose vaccination coverage was difficult to obtain in hard-to-reach areas in Papua (Indonesia’s easternmost region), since not all mothers can give birth in health facilities. 

#### 3.2.4. Workforce

Informants expressed the opinion that the ratio between health providers and the population was, at the moment, inadequate. They also reported that almost all health staff members have to handle two or even more different programmes, and are burdened with administrative jobs and field jobs as well, such as vaccination and health promotion. Informants said that the collaboration with other actors, such as community leaders, and religious leaders, is considered a good solution. For instance, some informants said that the health cadre helped to implement a vaccine schedule with reminders for their community. Moreover, informants also reported having limited staff and staff members who do not possess the appropriate competencies—as happens, for example when new staff for a particular programme lack the relevant background and experience. Therefore, they argued that it is necessary to conduct special training for new staff, as well as regular training for all staff.

#### 3.2.5. Tools

Informants in the interview reported that the dissemination of hepatitis B information currently mostly uses conventional tools, such as posters and billboards. However, informants felt that these approaches are ineffective due to the fact that most of the information given talks about the elimination of three diseases—hepatitis, AIDS, and syphilis—instead of discussing hepatitis B in detail. One other informant also said that information about hepatitis B using billboards was disseminated for particular events only, such as World Hepatitis Day, which is celebrated annually. Other than that, informants argued that posters distributed by the Ministry of Health did not reach many people, especially in rural areas, since most of the local population uses a local language instead of Bahasa, Indonesia’s national language. As a result, the posters’ message was not well understood by local communities.

In general, a variety of methods is used for the dissemination of health information. For instance, some informants reported that health staff directly disseminated information by, for example, using loudspeakers from the mosque or village hall, or even on the road. Health providers also conducted special meetings with religious leaders and community leaders to minimise hoax issues related to vaccination. Informants believed that this information could also be spread through regular community meetings, such as prayer recitations and Friday sermons, with the aim of informing the community about vaccine benefits and reminding it about the different vaccine schedules. They also believed that disseminating information through social media platforms (such as YouTube, Facebook, Instagram, and Twitter) and local culture-based dissemination, such as *Wayang Cakruk* (a regular traditional-puppets show in Gunungkidul), are also considered a potential medium for disseminating information that can reach different levels within a community.

### 3.3. Integrated Findings

There are six main pillars from the integrated qualitative and quantitative findings concerning the barriers and facilitators of hepatitis B vaccination on adults in Indonesia. For more details on the figure of Pillar Integration Process (see Table S4).

#### 3.3.1. Theme One: Cultural Norms of the Region

Our survey identified that vaccination coverage for hepatitis B was higher in Yogyakarta than in Aceh. This finding was supported by the qualitative result. For instance, almost all informants said that in Aceh, the halal controversy might be the reason people lack confidence in the vaccine, even when they know about its benefits. In addition, most of the informants said that communication through religious leaders and community leaders was a practical approach to disseminating information on vaccination.

#### 3.3.2. Theme Two: The Role of Health Care Workers

Our integrated findings showed that the role of health care workers was essential in vaccination uptake. In the quantitative arm, we found that most of the participants in our survey reported that they received hepatitis B information from a health provider, and this finding can be seen as a positive result related to health promotion and the dissemination of the hepatitis B vaccination. However, among health care workers in our survey, only 25.0% were vaccinated against hepatitis B. In addition, most informants highlighted that knowledge of hepatitis B infection and vaccination among health care workers is low, with many of them not being able to distinguish hepatitis B from other types of hepatitis, and not realising that they work with a high-risk population. Therefore, most of the informants suggested that regular training regarding hepatitis B infection and vaccination for health care workers is needed, including training on the effective dissemination of information to the community.

#### 3.3.3. Theme Three: Knowledge of Hepatitis B Infection and Vaccination

Findings regarding the knowledge of hepatitis B infection and vaccination converged in the quantitative and qualitative arms of our study. Our survey found that fewer than 20% of the participants had a good knowledge of hepatitis B infection and vaccination. This finding was supported by the qualitative results: Almost all of the informants argued that people’s understanding of hepatitis B is low. It is not surprising that some informants said people feel that vaccination is not important due to the fact that they do not know its benefits. 

#### 3.3.4. Theme Four: Accessibility of Hepatitis B Vaccination

The integrated results showed that insufficient accessibility of hepatitis B vaccination was one of the barriers to hepatitis B vaccination coverage in Indonesia. In the quantitative arm, 3.2% of unvaccinated survey participants said that the hepatitis B vaccination is unavailable at the nearest health facilities. This finding is supported by the qualitative results, with some informants complaining that obstacles in geographical access impede the implementation of vaccination programmes, especially among participants who live in rural areas, such as mountain areas.

#### 3.3.5. Theme Five: Affordability of Hepatitis B Vaccination

Another obstacle for vaccination coverage is its costs, as the vaccinations are not affordable for some people. In the quantitative arm, our survey identified that only 53.4% of participants were willing to pay for hepatitis B vaccination by themselves. Similarly, some informants in the qualitative study reported that hepatitis B vaccination was expensive, at IDR 200,000 to IDR 300,000 (USD 14 to 21) per dose. An informant said the price would be unaffordable for some people, especially those with low and irregular incomes. As a result, it is not surprising that 21.0% of unvaccinated survey participants reported that one of the most common reasons to not get vaccinated was the expense.

#### 3.3.6. Theme Six: The Dissemination of Information

Although our survey found that health providers and the media were the common sources of information about hepatitis B infection and vaccination, most informants in the qualitative study admitted that the available information regarding the hepatitis B programme was limited. Some informants reported that health officers provide health information to the community through direct health initiatives in various ways, but have limited human resources. Therefore, most informants suggested several potential interventions to increase public knowledge regarding hepatitis B by optimising information dissemination, such as culture and arts-based dissemination, religion-based dissemination, and an expanded use of social media.

## 4. Discussion

In this mixed methods study, we established six pillars that act as barriers to, and facilitators of, vaccine uptake: cultural norms of the regions, the role of the health care workers, knowledge of hepatitis B infection and vaccination, accessibility to hepatitis B vaccination, affordability of hepatitis B vaccination, and dissemination of information regarding hepatitis B infection and vaccination.

This study found that vaccination coverage for hepatitis B differs strongly by region, in this case, the province. This finding is associated with both provinces’ different histories, cultures, and local regulations. In Aceh, a province that applies Sharia law, vaccine rejection due to a vaccine ingredient that purportedly contains pork, and is therefore considered haram, is a common problem [23]. Meanwhile, Yogyakarta, a region that is defined by Javanese culture, does not have these problems [32], and has the highest vaccination coverage of existing vaccination programmes among children in Indonesia. These findings are in line with the results of a previous systematic review on hepatitis B vaccination in developing countries [30]. A previous study from Indonesia also found that geographic conditions contribute to disparities in the vaccination coverage because each region has its own political situation, religious affiliation, economic potential, and population density that impact the level of development of health programmes [33]. Drawing a conclusion from these findings, we want to highlight that there is no one-size-fits-all solution for all regions; thus, vaccination programmes should use culturally appropriate approaches and should be adapted based on local needs [34,35]. 

Another barrier is lack of accessibility. The low accessibility is connected to Indonesia’s geography, which consists of more than 16,000 islands [36]. Other studies have also found that low awareness of the availability of vaccinations occurs due to limited access to health facilities in some countries [15,37,38,39]. For instance, 80% of the non-vaccinated population in a hard-to-reach region in the UK declared that they had not been offered the vaccine, suggesting a lack of vaccine availability [40]. Even if misunderstandings and poor knowledge of services are well addressed, the situation will not effectively improve without addressing service accessibility [41,42].

Our study also found that hepatitis B vaccination is associated with exposure to accurate information related to hepatitis B infection, and the benefits of vaccination as one of the most effective preventive methods. Although information about hepatitis B and vaccination is available through the media, such as television, scientific magazines, and the internet [43], Njoroge et al. found that direct information from health providers was most closely associated with being vaccinated for hepatitis B [44]. However, some studies found that knowledge of hepatitis B and vaccination among health care workers is low [28,45,46,47]. For example, a study among nurses and midwives from Sudan found that the percentage of respondents with at least an average knowledge of hepatitis B virus was less than 60% [47].

Our survey results showed that most of the participants had a low knowledge about hepatitis B infection and vaccination. This finding was not surprising, considering that a previous study in developing countries reported similar findings [26]. Accurate information is expected to positively impact the relevant knowledge of hepatitis B infection and vaccination [48], and Lee et al. highlighted that missing information about vaccination could reduce people’s willingness to carry out hepatitis B vaccinations [39]. Some previous studies have claimed similar findings [26,49,50]. Hence, dissemination of information regarding vaccination also needs other support, such as that of religious leaders and community leaders, especially in reference to this sensitive issue [51,52,53]. For example, in Indonesia, a halal certification (*fatwa*) regarding the permissibility of the vaccination, provided by a council of religious scholars called *Majelis Ulama Indonesia*, plays an important role in community acceptance of vaccination [23,52]. When it comes to the regions of Indonesia, hepatitis B and vaccination information should also be adapted to the local conditions and culture to make it easier to understand and accept. For instance, reminder stickers positively affected vaccination coverage among children in another study in Indonesia [54].

Finally, this study also identified the vaccination cost as one barrier to hepatitis B vaccine uptake. Since hepatitis B vaccination is not mandatory for adults in Indonesia, people must actively decide to get immunised and privately cover the expenses for the vaccination costs [7]. For instance, informants of our qualitative study claimed that vaccination costs are one of the obstacles to achieving high vaccine coverage in Yogyakarta, which is known as a province with a lower minimum wage compared to Aceh. We calculated that vaccination takes up about 14% and 8% of the minimum monthly income in Yogyakarta and Aceh per dose, respectively [55]. 

The strengths of this study include the mixed method design, that described potential discrepancies and correspondences of barriers and facilitators from two different sides: the population (outpatient and health care worker) and the government. This method helps to increase the validity of findings. Nonetheless, this study has some limitations. First, more than 80% of the selected participants in the survey were women, which might reduce the generalisability of the study’s results. At the same time, the predominance of female participants is representative of the selected health care setting, since the community health centres provide health services targeting mothers and children, such as antenatal care and children’s vaccinations, to support the Indonesian Ministry of Health’s mandate to improve mother and child health services [56]. Second, due to missing data our survey could not investigate the economic factors that significantly impacted vaccination status in other studies [12,16,57]. The huge percentage of missing data on income might be explained by the fact that many people in Indonesia consider income very private. Lastly, due to time and resource constraints, our study was limited not only to outpatients and health care workers in the sixteen selected local health centers, but also limited to two provinces, with their own specific characteristics, which may differ from the overall population and the other 36 provinces in Indonesia. Although the findings may not be generalisable outside these two provinces, this study’s results can inform future vaccination intervention strategies and contribute to public health knowledge. 

## 5. Conclusions

Through integrated findings displaying the population’s and the government’s perspective, our study found that a lack of information impacted people’s knowledge regarding hepatitis B infection and vaccination. Further support from health providers and other stakeholders is needed to achieve high vaccination coverage through culturally appropriate approaches. Beyond that, vaccination access, including the availability and cost of vaccination, also plays an essential role in vaccine uptake. Further studies to explore those aspects among other stakeholders, such as local religious and community leaders or local health offices from other provinces, could be helpful for improving knowledge on the topic.

## Figures and Tables

**Table 1:** Factors associated with hepatitis B vaccination uptake (crude (Model 1) and multivariable logistic regression (Model 2)).

Variables	Vaccination Uptake n (%)	Model 1 COR (95% CI)	Model 2 AOR (95% CI)
Province			
Aceh	46 (33.8)	REF	REF
Yogyakarta	90 (66.2)	1.90 (1.30–2.79)	2.43 (1.56–3.78)
Age (years old)			
<35	54 (39.7)	REF	REF
35–50	59 (43.4)	2.49 (1.43–4.33)	0.96 (0.60–1.53)
>50	23 (16.9)	1.73 (1.00–3.01)	0.69 (0.35–1.35)
Sex			
Men	28 (20.6)	REF	REF
Women	108 (79.4)	0.87 (0.55–1.37)	1.22 (0.72–2.06)
Residency			
Urban	74 (54.4)	REF	REF
Rural	62 (45.6)	1.21 (0.84–1.74)	0.91 (0.59–1.40)
Marital status			
Single/Widowed/Divorced	21 (15.4)	REF	REF
Married	115 (84.6)	1.71 (1.04–2.80)	1.07 (0.57–2.00)
Religion			
Moslem	132 (97.1)	REF	REF
Christian and others	4 (2.9)	1.50 (0.52–4.32)	3.07 (0.94–10.01)
Education level			
Primary	2 (1.5)	REF	REF
Secondary	9 (6.6)	8.19 (4.09–16.43)	3.14 (1.38–7.15)
Tertiary	125 (91.9)	21.96 (5.36–89.97)	8.88 (1.96–40.21)
Occupational status			
Unemployed	5 (3.7)	REF	REF
Employed	131 (96.3)	11.58 (4.68–28.66)	1.04 (0.26–4.14)
Profession			
Non-Health care worker	9 (6.6)	REF	REF
Health care worker (low risk)	25 (18.4)	2.25 (1.48–3.65)	2.84 (1.66–4.86)
Health care worker (high risk)	102 (75.0)	17.44 (8.66–35.13)	5.74 (2.52–13.10)
Health insurance for vaccination			
No	71 (52.2)	REF	REF
Yes	65 (47.8)	6.46 (4.33–9.63)	4.11 (2.63–6.43)
Knowledge of hepatitis B			
Poor	10 (7.4)	REF	
Fair	64 (47.1)	2.79 (1.86–4.21)	
Good	62 (45.5)	34.05 (15.21–76.23)	
Risk perception of hepatitis B			
Low	31 (22.8)	REF	
High	105 (77.2)	3.34 (2.19–5.11)	
Willingness to be vaccinated			
No	5 (3.7)	REF	
Yes	131 (96.3)	4.79 (1.92–11.95)	

Model 1: Crude OR (COR). Model 2: Adjusted OR (AOR), Mutually adjusted for all variables in the table).

**Table 2:** Demographic characteristics.

Variable	n (%)
Age (years old)	
<35	446 (49.9)
35–50	357 (40.0)
>50	90 (10.1)
Sex	
Men	167 (18.7)
Women	726 (81.3)
Province	
Aceh	419 (46.9)
Yogyakarta	474 (53.1)
Residency	
Urban	443 (49.6)
Rural	450 (50.4)
Marital status	
Single/Widowed/Divorced	201 (22.5)
Married	692 (77.5)
Religion	
Moslem	856 (95.9)
Christian and others	37 (4.1)
Education level	
Primary	139 (15.6)
Secondary	239 (26.8)
Higher	515 (57.7)
Occupational status	
Unemployed	237 (26.5)
Employed	656 (73.5)
Monthly income (IDR)	
≤1 million	43(4.8)
>1 to 2 million	27 (3.0)
>2 to 3 million	59 (6.6)
>3 million	33 (3.7)
Missing	731 (81.9)
Profession	
Non-medical health centre staff	386 (43.2)
Health care worker (low risk) ^†^	160 (17.9)
Health care worker (high risk) ^††^	347 (38.9)
Health insurance for vaccination	
No	734 (82.2)
Yes	159 (17.8)

^†^ Healthcare workers who work in low-risk unit, such as administrative personnel, pharmacy staff, and driver. ^††^ Healthcare workers who work in high-risk unit, such as medical doctor, nurse, midwife, and analyst.

**Table 3:** Knowledge, risk perception, willingness to pay, and vaccination status.

Variables	All Participants n = 893	HCW in High-Risk Unit n = 347	HCW in Low-Risk Unit n = 160	Non HCW n = 386
Knowledge of hepatitis B				
Score of knowledge related to hepatitis B infection and vaccination (Mean [95% Confidence Interval])	11.01 [10.65–11.37]	20.36 [19.92–20.80]	18.40 [17.73–19.07]	9.08 [8.46–9.72]
Knowledge concerning hepatitis B infection				
Poor	377 (42.2)	30 (8.6)	28 (17.5)	319 (82.6)
Fair	342 (38.3)	189 (54.5)	92 (57.5)	61 (15.8)
Good	174 (19.5)	128 (36.9)	40 (25.0)	6 (1.6)
Knowledge concerning hepatitis B vaccination				
Poor	350 (39.2)	50 (14.4)	38 (23.8)	262 (67.8)
Fair	371 (41.5)	166 (47.8)	87 (54.4)	118 (30.6)
Good	172 (19.3)	131 (37.8)	35 (21.8)	6 (1.6)
Risk perception of hepatitis B infection				
The score of risk perception related to hepatitis B infection (Mean ± 95% Confidence Interval)	12.59 [12.45–12.74]	13.44 [13.26–13.62]	13.00 [12.72–13.28]	11.65 [11.41–11.89]
Low	407 (45.6)	98 (28.8)	67 (41.9)	242 (62.7)
High	486 (54.4)	249 (71.8)	93 (58.1)	144 (37.3)
Willingness to get vaccinated *	(n = 757)	(n = 245)	(n = 135)	(n = 377)
No	117 (15.5)	14 (5.7)	16 (11.9)	87 (23.1)
Yes	640 (84.5)	231 (94.3)	119 (88.1)	296 (76.9)
Vaccination status				
Not vaccinated	757 (84.8)	245 (70.6)	135 (84.4)	377 (97.7)
Vaccinated	136 (15.2)	102 (29.4)	25 (15.6)	9 (2.3)

* Includes only those respondents who were not vaccinated at the time of the survey. HCW = Health Care Worker.

**Table 4:** Exposure to information regarding hepatitis B.

Variables	All Participants n = 893	HCW in High-Risk Unit n = 347	HCW in Low-Risk Unit n = 160	Non HCW n = 386
Heard about hepatitis B vaccination				
Yes	729 (81.6)	340 (98.0)	152 (95.0)	237 (61.4)
No	164 (18.4)	7 (2.0)	8 (5.0)	149 (38.6)
Source of information * ^a^	(n = 729)	(n = 340)	(n = 152)	(n = 237)
Health provider	527 (72.3)	289 (85.0)	125 (82.2)	113 (47.7)
Health community	34 (4.7)	11 (3.2)	5 (3.3)	18 (7.6)
Family/relatives	161 (22.1)	93 (27.4)	49 (32.2)	19 (8.0)
Community/Religious leaders	17 (2.3)	10 (2.9)	3 (2.0)	4 (1.7)
Media	397 (54.5)	229 (67.4)	108 (71.1)	60 (25.3)
Seminar/Training	43 (5.9)	25 (7.4)	3 (2.0)	15 (6.3)
Media platform ** ^b^	(n = 397)	(n = 229)	(n = 108)	(n = 60)
Television	251 (63.2)	153 (66.8)	73 (67.6)	25 (41.7)
Social media	215 (54.2)	146 (63.8)	59 (54.6)	10 (16.7)
Poster	88 (22.2)	63 (27.5)	18 (16.7)	7 (11.7)
Newspaper	63 (15.9)	41 (17.9)	17 (15.7)	5 (8.3)
Radio	27 (6.8)	22 (9.6)	5 (4.6)	0 (0.0)
Internet	17 (4.3)	8 (3.5)	4 (3.7)	5 (8.3)

HCW = Health Care Worker. * Includes only those respondents who had ever heard about hepatitis B vaccination at the time of the survey. ** Includes only those respondents who received information of hepatitis B vaccination from media platform at the time of the survey. ^a^ participant had more than one source of information. ^b^ participant used more than one media platform.

**Table 5:** The themes, subthemes, and representative quotes.

Theme	Subthemes	Representative Quote(s)
Population	Knowledge	‘For existing programmes (child vaccination), there is a rejection from a particular group. They said vaccination is not important. This is due to a lack of knowledge and awareness about vaccination. In addition, some mothers were afraid of the side effects of vaccination, such as fever’ (Transcript 11).
	Cultural norms and vaccine controversy	‘There are cultural differences between Aceh and Yogyakarta (which affect the vaccination program). For example, the vaccination (coverage) of children in Aceh is low because of public perceptions. They feel that people health is a gift from God. In addition, the controversy about the issue of Halal and Haram related to vaccine ingredients (containing pork) or Jewish conspiracies. All of these issues impacted vaccination coverage’ (Transcript 4). In my opinion, people in Gunungkidul and are still easily driven by certain figures. We still have a king. If the king says, we are still obedient to carry out it. But, I think it’s not the same with outside Yogyakarta province. Our society is still easy to move, believe in the leadership, and easy to be directed to in accordance with government programmes (Transcript 7).
Governance	Policies: regulation	‘Currently, the hepatitis B vaccination programme provided by the government is still limited to children, and its coverage is still low...where most of the adult population is susceptible to hepatitis B infection. Therefore, further vaccination is needed for the adult population in Indonesia; at least the community knows about hepatitis B vaccination for adults’ and can access it’ (Transcript 5).
	Financing	‘Currently, Indonesia has two vaccination programmes, namely the national and elective programmes. Hepatitis B vaccination for adults is an optional programme. So, for adults who want to receive hepatitis B vaccination, they seek vaccination and pay for it themselves’ (Transcript 2).
	Insurance and purchasing	
	Trustworthy data	‘Yes indeed, this is some of the homework that we must do. That is true; our data (related to hepatitis B) are still minimal. So that is why there are recommendations in our planning from the committee expert, one our target (has to improve) is reporting’ (Transcript 12).
Platforms	Geographic access to facilities	‘As far as I know, there are some hard-to-reach areas in Indonesia that we (MoH in Jakarta) might be unable to monitor very well. For example, in Papua, implementing a hepatitis B birth dose (HB0) that should be given less than 24 h might be difficult due to geographic conditions. Some gave HB0 seven days after delivery’ (Transcript 2).
Workforce	Managers: number and distribution	‘We have a lack of human resources. Most of them handle more than one programme. For example, surveillance and Hajj. In addition, some of our staff have already retired’ (Transcript 11).
	Skill	‘There is no other staff with a health promotion background in Gunungkidul Regency except me. So, this can be an obstacle to improving our programme. In addition, we only have one staff member for administration and data analysis. We need more staff with the proper skill to achieve our programme’ (Transcript 7).
	Teamwork	‘We collaborated with other sectors. Usually, we involve the Ministry of Religion, Youth and Sports Office and cadres to educate the community about the benefit of vaccination and vaccine implementation’ (Transcript 11).
Tools	Hardware: equipment	‘We need a tool for disseminating information that uses the local language so that local people can understand the message’ (Transcript 3).
	Information system	‘We would like to develop culture-based dissemination, for example, Wayang cakruk. This medium will reach many layers in our community. It still really likes this Wayang segmentation. We believe that the traditions that exist and only exist in Gunungkidul can convey the message from us (health providers). However, this is an expensive medium due to its use of many instruments’ (Transcript 7).

## Data Availability

The data presented in this study are available on request from the first author.

## Supplementary Materials

The following supporting information can be downloaded at: https://www.mdpi.com/article/10.3390/vaccines11020398/s1, Table S1: Questionnaire Survey of Knowledge, awareness, risk perception, attitudes, practice and willingness of Hepatitis B virus infection and vaccination among outpatients and health care workers in Indonesia 2020; Table S2: Question list and proportion of correct answer of knowledge regarding hepatitis B infection and vaccination; Table S3: Question list and proportion of high risk-perception regarding hepatitis B infection and vaccination. Table S4: Pillar integration process.
