# Supplementary material for: Barriers to and Facilitators of Hepatitis B Vaccination among the Adult Population in Indonesia: A Mixed Methods Study
Source: Vaccines (Basel). 2023 Feb 9;11(2):398. doi: 10.3390/vaccines11020398 (PMC9967628; doi:10.3390/vaccines11020398)
Supplement: Supplementary file 1 [file vaccines-11-00398-s001.zip › vaccines-2189639-supplementary.pdf]

Table S1. Questionnaire Survey of Knowledge, awareness, risk perception, attitudes, practice and willingness of Hepatitis B virus infection (HBV) and vaccination among outpatients and health care workers in Indonesia 2020.

	QUESTION		CODING
**A**	**GENERAL INFORMATION**		
A1.	Name of Province	1. Aceh<br>2. Yogyakarta	
A2.	Urban/rural	1. Urban<br>2. Rural	
A3.	Name of City/District	1. Banda Aceh<br>2. Gayo Lues<br>3. Kota Yogyakarta<br>4. Gunungkidul	
A4.	Name of Health centre	1. Banda Raya (Mibo)<br>2. Kuta Alam<br>3. Lampulo<br>4. Kopelma Darrusalam<br>5. Pintu Rime Uring<br>6. Rerebe<br>7. Blang Jerango<br>8. Terangon<br>9. Tegalrejo<br>10. Danurejan II<br>11. Umbulharjo I<br>12. Wirobrajan<br>13. Nglipae I<br>14. Patuk II<br>15. Ponjong I<br>16. Ngawen II	
A5.	Date of inteview	(DD/MM/YYYY)	__/__/____
A6.	Time of interview (Start)	WIB	
A7.	Time of inteview (End)	WIB	
A8.	Name of interviewer		
A9.	Sign of interviewer		
**B**	**DATA ENTRY**		
B1.	Date of check by investigator/Assistance	(DD/MM/YYYY)	__/__/____
B2.	Sign of investigator		
B3.	Date of entry	(DD/MM/YYYY)	__/__/____
B4.	Name of data entry		
B5.	Sign of data entry		
**C**	**CHARACTERISTIC OF RESPONDENCE**		

	QUESTION		CODING
C1.	Name of respondence		
C2.	Sex	1. Male<br>2. Female	
C3.	Date of birth –**(Check the ID, if need it)**	(DD/MM/YYYY)	__/__/____
C4.	Age	In year	
C5.	Ethnic	1. Aceh/Achin/Akhir/Asji/A-Tse/Ureung Aceh<br>2. Alas, Aneuk Jamee,Gayo,<br>3. Gayo Lut,<br>4. Gayo Luwes,<br>5. Gayo Serbe Jadi,<br>6. Kluet,<br>7. Sigulai,<br>8. Simeulue,<br>9. Singkil,<br>10. Tamiang<br>11. Batak<br>12. Jawa<br>13. Melayu<br>14. Minang<br>15. Sunda<br>16. Madura<br>17. Betawi<br>18. Lampung<br>19. Serawai (Bengkulu)<br>20. Bugis<br>21. Nias<br>22. Lainnya, sebutkan:________________	
C6.	Education	1. Never school<br>2. Ungraduate of primary school<br>3. Primary school<br>4. Secondary school<br>5. High school<br>6. Academi/Diploma<br>7. Bachelor/Master/Doctoral	
C7.	Marital status	1. Single<br>2. Marriage<br>3. Divorced<br>4. Widowed	
C10	Status of respondence in this tsudy<br>**(fill by Enumerator)**	1. Patient / Non Health worker<br>2. Health care worker – **Jump to C12**	
C11	Reason to visit this health care	1. Treatment<br>2. Medical check up<br>3. Vaccination<br>4. Reguler visite (including take the Tuberculosis drug)<br>5. Others: ________________	
C12	**For respondence who answear C10=2 (health care worker)**<br>Role of respondence in this health care	1. Phsician<br>2. Dentist<br>3. Nurse<br>4. Midwife<br>5. Pharmacist	

	QUESTION			CODING
		6. Radiologist<br>7. Analist<br>8. Others:____________________		
C13	Unit of work	1. Emergency<br>2. Intern<br>3. Dentist<br>4. Children<br>5. Obgyn<br>6. Pharmacy<br>7. Laboratory<br>8. Radiology<br>9. Radiologi<br>10. Others:____________________		
C14	How long you have been work ini this health care	In year	________________year	
C15	Income in a month (avarage)	In Rupiah	Rp.	
C16	Expenditure in a month (avarage)	In Rupiah	Rp.	
**D**	**PREVIOUS EXPOSURE—both patient and health care worker**			
D1	Have you ever had any surgery?	1. Yes (one time)<br>2. Yes (more than one time/often)<br>3. No -— **Jump to D3**		
D2	When did you had the surgery? (If more than one times, show **the newest** surgery)	MM/YYYY		__/____
D3	Have you ever had blood transfussion?	1. Yes (one time)<br>2. Yes (more than one time/often)<br>3. No -— **Jump to D5**		
D4	When did you had the blood transfussion? (If more than one times, show **the newest** blood transfussion)	MM/YYYY		__/____
D5	Have you ever got injection treatment in Hospital or other places?	1. Yes (one time)<br>2. Yes (more than one time/often)<br>3. No -— **Jump to D8**		
D6	When did you had the blood transfussion? (If more than one times, show **the newest** injection treatment)	MM/YYYY		__/____
D7	Where did you got the injection treatment?	1. Hospital/Klinik dokter/Puskesmas<br>2. Beauty care<br>3. Others, ______________		
D8	Have you ever got needle treatment?	1. Yes (one time)<br>2. Yes (more than one time/often)<br>3. No -— **Jump to D11**		
D9	When did you had the needle treatment? (If more than one times, show **the newest** needle treatment)	MM/YYYY		__/____
D10	What kind of the needle treatment?	1. Acupunture<br>2. Bekam<br>3. Others, ______________		

	QUESTION		CODING
D11	Have you ever been hospitalized for more than three days?	1. Yes (one time)<br>2. Yes (more than one time/often)<br>3. No --- **Jump to D14**	
D12	When did you had the hospitalized? (If more than one times, show **the newest** hospitalized)	MM/YYYY	__/____
D13	How long you have been hospitalized?	Days	________________days
D14	Have you ever had dental treatment?	1. Yes (one time)<br>2. Yes (more than one time/often)<br>3. No --- **Jump to D16**	
D15	When did you had the dental treatment? (If more than one times, show **the newest** dental treatment)	MM/YYYY	__/____
D16	Have you ever had body part pierced?	1. Yes (one time)<br>2. Yes (more than one time/often)<br>3. No --- **Jump to D18**	
D17	When did you had the body part pierced? (If more than one times, show **the newest** body part pierced)	MM/YYYY	__/____
D18	Have you ever had tatoo?	1. Yes (one time)<br>2. Yes (more than one time/often)<br>3. No --- **Jump to D20**	
D19	When did you had the tatoo? (If more than one times, show **the newest** shared)	MM/YYYY	__/____
D20	Have you ever shared toorhbrush?	1. Yes (one time)<br>2. Yes (more than one time/often)<br>3. No --- **Jump to D22**	
D21	When did you had the toothbrush? (If more than one times, show **the newest** shared)	MM/YYYY	__/____
D22	Have you ever shared Razor?	1. Yes (one time)<br>2. Yes (more than one time/often)<br>3. No --- **Jump to D24**	
D23	When did you had the Razor? (If more than one times, show **the newest** shared)	MM/YYYY	__/____
D24	Do you have a family member with Hepatitis B?	1. Yes<br>2. No<br>3. Do not know	
D25	Do you have a friend with Hepatiis?	1. Yes<br>2. No<br>3. Do not know	
**Question for Health care worker only**			
D26	Have you ever had history of exposure to blood or body fluids on intact skin in the last one year?	1. Yes<br>2. No<br>3. Do not know/remember	
D27	Have you ever had history of splash of blood or body fluids to eye or mouth in the last one year?	1. Yes<br>2. No<br>3. Do not know/remember	
D28	Have you ever had history of splash of blood on cuts or unprotected skin in the last one year?	1. Yes<br>2. No	

	QUESTION		CODING
		3. Do not know/remember	
D29	Have you had a needle stick injury in the last one year?	1. Yes<br>2. No<br>3. Do not know/remember	
**E**	**SOURCE OF INFORMATION ABOUT HEPATITIS B AND VACCINATION**		
E1	Have you ever heard about Hepatitis B infection/disease?	1. Yes<br>2. No --- **Jump to E4**	
E2	Where have you heard the information about Hepatitis B infection/disease?<br>**(Multiple answear, write'1' if you choose it and 0 if not in the coding box)**	Health care provider/Physician	
		Family members	
		Community function or Health fairs	
		Religious leaders	
		Friends	
		Employee asistance programs	
		Co-workers	
		Traditional healers	
		Media (TV, Socialmedia, Newspaper, Banner, Poster, etc)	
		Others:___________________________	
E3	If the answear is MEDIA, What kind of media that you got the information abaut Hepatitis B infection?<br>**(Multiple answear, write'1' if you choose it and 0 if not in the coding box)**	TV	
		Social media :_________	
		Poster in:_____________	
		Newspaper	
		Radio	
		Others:_______________	
E4	Have you ever heard about Hepatitis B Vaccination for adult?	1. Yes<br>2. No --- **Jump to F**	
E5	Where have you heard the information about Hepatitis B Vaccination<br>**(Multiple answear, write'1' if you choose it and 0 if not in the coding box)**	Health care provider/Physician	
		Family members	
		Community function or Health fairs	
		Religious leaders	
		Friends	

	QUESTION		CODING
		Employee asistance programs	
		Co-workers	
		Traditional healers	
		Media (TV, Socialmedia, Newspaper, Banner, Poster, etc)	
		Others:___________________________	
E6	If the answear is MEDIA, What kind of media that you got the information abaut Hepatitis B vaccination? **(Multiple answear, write'1' if you choose it and 0 if not in the coding box)**	TV	
		Social media :_________	
		Poster in:_____________	
		Newspaper	
		Radio	
		Others:_______________	
E7	In your oponion, which source that can influence more people to known well about Hepatitis B infection and vaccination? **(choose the best three – sort from 1 to 3 from the best based on your opinion)**	Doctor/family doctor	
		Parents/Family	
		Friends	
		Lead og religion (Ustad, Pastur, etc)	
		Lead of village (Kades,Kadus,etc)	
		Public health official/facilities (Puskesmas, Hospital)	
		Public helath websites	
		Other websites	
		Social media (Facebook, Twitter, Instagram, Youtube,etc)	
		Poster in public area	
		Other: ___________________	
**F**	**KNOWLEDGE OF HEPATITIC B INFECTION**		
F1	Hepatitis B caused by bacteria	1. True<br>2. False<br>3. Do not know	
F2	Hepatitis B can be transmitted through food or drink	1. True<br>2. False<br>3. Do not know	

	QUESTION		CODING
F3	Hepatitis B can be transmitted through blood/blood product	1. True<br>2. False<br>3. Do not know	
F4	Hepatitis B can be transmitted through sexual intercourse	1. True<br>2. False<br>3. Do not know	
F5	Hepatitis B can be transmitted through sharing use of needles	1. True<br>2. False<br>3. Do not know	
F6	Hepatitis B can be transmitted through mother to infant	1. True<br>2. False<br>3. Do not know	
F7	Hepatitis B can be transmitted through shaking hand with infected person	1. True<br>2. False<br>3. Do not know	
F8	Hepatitis B can be transmitted through sharing toothbrush with an infected person	1. True<br>2. False<br>3. Do not know	
F9	Hepatitis B can be transmitted through being coughed on by an infected person	1. True<br>2. False<br>3. Do not know	
F10	Hepatitis B infection can be transmitted to your partner	1. True<br>2. False<br>3. Do not know	
F11	Hepatitis B can be prevent by hepatitis B vaccination	1. True<br>2. False<br>3. Do not know	
F12	Hepatitis B can be prevent byregular exercise	1. True<br>2. False<br>3. Do not know	
F13	Hepatitis B can be prevent by balanced diet	1. True<br>2. False<br>3. Do not know	
F14	Hepatitis B can be prevent by good hand hygiene	1. True<br>2. False<br>3. Do not know	
F15	Hepatitis B can be prevent by use of condom	1. True<br>2. False<br>3. Do not know	
F16	There is blood screening for hepatitis B infection	1. True<br>2. False<br>3. Do not know	
F17	There is antiviral therapy for hepatitis B infection	1. True<br>2. False<br>3. Do not know	
F18	Hepatitis B can be cured	1. True<br>2. False<br>3. Do not know	

	QUESTION		CODING
F19	Hepatitis B is a risk factor for cirrosis	1. True<br>2. False<br>3. Do not know	
F20	Hepatitis B is a risk factor for liver cancer	1. True<br>2. False<br>3. Do not know	
**G**	**KNOWLEDGE OF HEPATITIS B VACCINATION**		
G1	There is hepatitis B vaccine available for those who are not immune	1. True<br>2. False<br>3. Do not know	
G2	Hepatitis B vaccination is not for all people	1. True<br>2. False<br>3. Do not know	
G3	Hepatitis B vaccination is contraindicated in pregnancy	1. True<br>2. False<br>3. Do not know	
G4	Hepatitis B vaccination can be given as post-exposure prophylaxis	1. True<br>2. False<br>3. Do not know	
G5	Hepatitis B vaccination does not increase the risk for complication	1. True<br>2. False<br>3. Do not know	
G6	Neonatal Hepatitis B vaccination can prevent the transmission of Hepatitis B infection from mother to babies	1. True<br>2. False<br>3. Do not know	
G7	Indonesia government is providing free neonatal hepatitis B vaccination for new born babies	1. True<br>2. False<br>3. Do not know	
G8	Indonesia government is providing free hepatitis B vaccination for adult	1. True<br>2. False<br>3. Do not know	
G9	Hepatitis B vaccination is effective to treat patients with acute Hepatitis B infection	1. True<br>2. False<br>3. Do not know	
**H**	**RISK PERCEPTION OF HEPATITIS B**		
H1	I have a high chance to get hepatitis B infection	1. Agree<br>2. Disagree	
H2	I can get seriously ill from the hepatitis B vaccine	1. Agree<br>2. Disagree	
H3	Hepatitis B infection is so rare that I do not need to be immunized against it	1. Agree<br>2. Disagree	
H4	I never worried to be infected with hepatitis B	1. Agree<br>2. Disagree	
H5	Taking the hepatitis B screening is important to me	1. Agree<br>2. Disagree	
H6	I believe in hepatitis B vaccination regarding efficacy	1. Agree<br>2. Disagree	
H7	If I am not vaccinated with hepatitis B vaccine, I am at significant risk for getting hepatitis B	1. Agree<br>2. Disagree	

	QUESTION		CODING
	infection		
H8	It is better to develop the immunity by getting vaccinated	1. Agree<br>2. Disagree	
H9	The hepatitis B vaccination is safe	1. Agree<br>2. Disagree	
H10	I can get seriously ill from the hepatitis B vaccine	1. Agree<br>2. Disagree	
H11	Receiving the hepatitis B vaccine will protect the people around me from getting hepatitis B infection	1. Agree<br>2. Disagree	
H12	Receiving the hepatitis B vaccine is important to me	1. Agree<br>2. Disagree	
H13	I am not really sure to take hepatitis B vaccination	1. Agree<br>2. Disagree	
H14	Generally other people around me view vaccination favourably	1. Agree<br>2. Disagree	
H15	I think, hepatitis B vaccine will not prevent me from hepatitis B infection	1. Agree<br>2. Disagree	
**I**	**ATTITUDES OF HEPATITIS B TRANSMISSION (Health Care Worker only)**		
I1	Do you dispose sharp objects in the sharp box?	1. Always<br>2. Sometimes<br>3. Never	
I2	Do you recap needle before disposing?	1. Always<br>2. Sometimes<br>3. Never	
I3	Do you prevent injuries when using sharp devices?	1. Sure<br>2. Not Sure<br>3. No	
I4	Do you ensure safe clinical waste management?	1. Always<br>2. Sometimes<br>3. Never	
I5	Do you wear gloves before taking blood samples and other body fluids from patients?	1. Always<br>2. Sometimes<br>3. Never	
I6	Do you change your gloves in between patients?	1. Always<br>2. Sometimes<br>3. Never	
I7	Do you clean your hand properly after each patients?	1. Always<br>2. Sometimes<br>3. Never	
I8	Do you wear ward coats?	1. Always<br>2. Sometimes<br>3. Never	
I9	Do you disinfected reusable equipment?	1. Always<br>2. Sometimes<br>3. Never	
I10	Do you ensure use of adequate procedure for routine cleaning?	1. Always<br>2. Sometimes<br>3. Never	
**J**	**PRACTICE OF HEPATITIS B VACCINATION**		
J1	Are you vaccinated for Hepatitis B?	1. Yes<br>2. No –**Jump to J3**	
J2	Are you fully vaccinated (3 doses)?	1. Yes<br>2. No	

	QUESTION		CODING
J3	Have you been screened/test for Hepatitis B?	1. Yes<br>2. No	
J4	Is there your family member vaccinated for Hepatitis B?	1. Yes<br>2. None<br>3. Do not know	
J5	Which of your family members are vaccinated for Hepatitis B?<br>**(Multiple answear, write'1' if you choose it and 0 if not in the coding box)**	Father	
		Mother	
		Sibling	
		Husband/Wife	
		Children	
		Others:___________________	
**K**	**REASON FOR UPTAKE HEPATITIS B VACCINATION**		
	**For respondence who answear J1=1 (YES)**<br><br>**(choose the best three – sort from 1 to 3 from the best based on your opinion)**	Physician/Nurse/Health worker advised me	
		Concern abaut my future health	
		I have family member with Hepatitis B disease	
		My spouse is Hepatitis B patient	
		My job is high potential to infected	
		Mandatory from my company (work)	
		Others:___________________________	
**L**	**REASON FOR NOT VACCINATION**		
	**For respondence who answear J1=2 (NO)**<br><br>**(choose the best Five – sort from 1 to 5 from the best based on your opinion)**	I never felt need to vaccine	
		I never though to uptake hepatitis B vaccination before	
		I have never heard about the vaccine before/not enough information to make an informed decision	
		I do not believe on efectiveness of hepatitis B vaccination	
		I do not believe the safety of the vaccine/worry about side effect	
		The vaccine is not available at health facilities near me	
		I have not been offered a chance for Hepatitis B vaccination/ Vaccination fees are high	
		I am fear from injetion	
		I have no time for Hepatitis B vacination, very busy schedule	
		There is no enough education or informtaion concerning Hepatitis B	
		I can not afford the hepatitis B vaccination	

	QUESTION		CODING
		It is too far from my home / inconvenience due to distance	
		Someone advise me not to uptake the vaccination	
		Forbiden in my religion (haram)	
		Dont know where to be vaccine	
		Others: ___________________________	
**M**	**WILLINGNESS TO UPTAKE HEPATITIS B VACCINATION**		
M1	Have you ever come to health facilities to find hepatitis B vaccination information?	1. Yes<br>2. No	
M2	Have you ever come to health facilities to find hepatitis B vaccination services?	1. Yes<br>2. No	
M3	Do you have the current access of hepatitis B vaccination insurance?	1. Yes<br>2. No	
M4	Have you ever found the access of hepatitis B vaccination insurance?	1. Yes<br>2. No	
M5	Do you have willingness to take Hepatitis B vaccination?	1. Yes<br>2. No --– **Jump to M7**	
M6	How willing would you to get Hepatitis B Vaccination?	1. Very willing<br>2. Willing<br>3. Somewhat willing<br>4. Not at all willing	
M7	Do you have willingness to take hepatitis B vaccination if government provide free for adults?	5. Yes<br>6. No	
M8	Do you have willingness to take hepatitis B vaccination if BPJS cover the payment?	1. Yes<br>2. No	
M9	Will you pay to hepatitis B vaccination?	1. Yes<br>2. No → **FINISH**	
M10	How much that you will to pay for three doses of hepatitis B?	In Rupiah	Rp.________________ for 3 doses

Table S2. Question list and proportion of correct answer of knowledge regarding hepatitis B infection and vaccination

No	Questions	All Participants (n =757)	Aceh (n = 373)	Yogyakarta (n = 384)
Hepatitis B infection				
1	Hepatitis B is caused by bacteria	282 (37.3)	135 (36.2)	147 (38.3)
2	Hepatitis B can be transmitted through food or drink	264 (34.9)	110 (29.5)	154 (40.1)
3	Hepatitis B can be transmitted through blood/blood products	529 (69.9)	256 (68.6)	273 (71.1)
4	Hepatitis B can be transmitted through sexual intercourse	413 (54.6)	207 (55.5)	206 (53.6)
5	Hepatitis B can be transmitted through shared use of needles	567 (74.9)	255 (68.4)	312 (81.2)
6	Hepatitis B can be transmitted from mother to infant	511 (67.5)	246 (66.0)	265 (69.0)
7	Hepatitis B can be transmitted through shaking hands with an infected person	420 (55.5)	134 (35.9)	28.6 (74.5)
8	Hepatitis B can be transmitted through sharing a toothbrush with an infected person	488 (64.5)	231 (61.9)	257 (66.9)
9	Hepatitis B can be transmitted through being coughed on by an infected person	248 (32.8)	93 (24.9)	155 (40.4)
10	Hepatitis B infection can be transmitted to your partner	428 (56.5)	213 (57.1)	215 (56.0)
11	Hepatitis B can be prevented by hepatitis B vaccination	621 (82.0)	278 (73.7)	346 (90.1)
12	Hepatitis B can be prevented by regular exercise	161 (21.3)	71 (19.0)	90 (23.4)

13	Hepatitis B can be prevented by a balanced diet	194 (25.6)	81 (21.7)	113 (29.4)
14	Hepatitis B can be prevented by hand hygiene	68 (9.0)	26 (7.0)	42 (10.9)
15	Hepatitis B can be prevented by using a condom during sexual activity	411 (54.3)	181 (48.5)	230 (59.9)
16	There is blood screening for hepatitis B infection	615 (79.9)	271 (72.7)	334 (87.0)
17	There is antiviral therapy for hepatitis B infection	500 (66.1)	229 (61.4)	271 (70.6)
18	Hepatitis B can be cured	66 (8.7)	18 (4.8)	48 (12.5)
19	Hepatitis B is a risk factor for cirrhosis	530 (70.0)	251 (67.3)	279 (72.7)
20	Hepatitis B is a risk factor for liver cancer	446 (58.9)	226 (60.6)	220 (57.3)
Hepatitis B vaccination				
1	There is a hepatitis B vaccine available for those who are not immune	523 (69.1)	228 (61.1)	295 (76.8)
2	Hepatitis B vaccination is not for all people	307 (40.6)	142 (38.1)	165 (43.0)
3	Hepatitis B vaccination is contraindicated in pregnancy	166 (21.9)	88 (23.6)	78 (20.3)
4	Hepatitis B vaccination can be given as post-exposure prophylaxis	174 (23.0)	71 (19.0)	103 (26.8)
5	Hepatitis B vaccination does not increase the risk for complication	295 (39.0)	133 (35.7)	162 (42.2)
6	Neonatal Hepatitis B vaccination can prevent the transmission of Hepatitis B infection from mother to babies	466 (61.6)	209 (56.0)	257 (66.9)
7	Indonesian government is providing free neonatal hepatitis B vaccinations to new	536 (70.8)	269 (72.1)	267 (69.5)

	born babies			
8	Indonesias government is providing free hepatitis B vaccination for the adult population	187 (24.7)	72 (19.3)	115 (29.9)
9	Hepatitis B vaccination is effective for treatment of patients with acute Hepatitis B infection	195 (25.8)	76 (20.4)	119 (31.0)

Data presented as numbers (%)

Table S3. Question list and proportion of high risk-perception regarding hepatitis B infection and vaccination

No	Questions	All Participants (n =757)	Aceh (n = 373)	Yogyakarta (n = 384)
1	I have a high chance to get hepatitis B infection	373 (49.3)	194 (52.0)	179 (46.6)
2	I can get seriously ill from the hepatitis B vaccine	683 (90.2)	323 (86.6)	360 (93.8)
3	Hepatitis B infection is so rare that I do not need to be immunized against it	615 (81.2)	294 (78.8)	321 (83.6)
4	I never worried to be infected with hepatitis B	606 (80.1)	288 (77.2)	318 (82.8)
5	Taking the hepatitis B screening is important to me	695 (91.8)	334 (89.5)	361 (94.0)
6	I believe in hepatitis B vaccination regarding efficacy	682 (90.1)	311 (83.4)	371 (96.6)
7	If I am not vaccinated with hepatitis B vaccine, I am at significant risk for getting hepatitis B infection	570 (75.3)	293 (78.6)	277 (72.1)
8	It is better to develop the immunity by getting vaccinated	710 (93.8)	343 (92.0)	367 (95.6)
9	The hepatitis B vaccination is safe	64.5 (85.2)	305 (81.8)	340 (88.5)
10	I can get seriously ill from the hepatitis B vaccine	620 (81.9)	296 (79.4)	324 (84.4)
11	Receiving the hepatitis B vaccine will protect the people around me from getting hepatitis B infection	609 (80.4)	288 (77.2)	321 (83.6)
12	Receiving the hepatitis B vaccine is important to me	722 (95.4)	350 (93.8)	372 (96.9)

13	I am not really sure to take hepatitis B vaccination	746 (98.5)	365 (97.9)	381 (99.2)
14	Generally other people around me view vaccination favourably	681 (76.8)	268 (71.8)	313 (81.5)
15	I think, hepatitis B vaccine will not prevent me from hepatitis B infection	582 (76.9)	263 (70.5)	319 (83.1)

Data presented as number (%)

Table S4. Pillar integration process

Data	Categories		Themes	Verbatim
Province: These data represent the differences in issues between Aceh and Yogyakarta related to hepatitis B vaccination	Participants who lived in Yogyakarta were two times (AOR =2.30; 95% CI: 1.45–3.56) more likely to be vaccinated with hepatitis B vaccination than those who lived in Aceh.	Character and cultural norms between regions	People's beliefs	'This is related to culture, where the people (who live in Banda Aceh) are not indigenous people but come from outside (other cities/districts in Aceh). So, they have their own beliefs and need more information (regarding hepatitis B infection and vaccination)' (Transcript 5).
	There were 26 (3.43%) unvaccinated participants who claimed that vaccination is forbidden by their religion (Haram).		Cultural norms	'The majority of the people living in Aceh are Muslim. So, when we talk about vaccination, the general issue is related to the halal and haram of vaccination materials. They ask about '*Fatwa*' (Rules), the first question: *Halal* or *Haram* (Forbidden). Sometimes, even though the vaccines have a halal certificate, they are still rejected because of their own culture' (Transcript 5).
			Ways to correct perception	'Usually, we invite community leaders, religious leaders, and university speakers (experts) to talk about the vaccination program. In this meeting, we explain the benefits of vaccination from a health and religious perspective and straighten out negative rumours about vaccination, so that it can help spread valid information to the public through its role in society' (Transcript 11).

Profession	The participants who worked as health care workers in low-risk and high-risk units of hepatitis B were two (AOR = 2.73; 95% CI: 1.60–4.65) and three times (AOR = 5.70; 95% CI: 2.49–13.05) more likely to accept hepatitis B vaccination than those who were not health care workers.	Role of the health care worker in the population	Knowledge of hepatitis B infection and vaccination among health care workers is low.	'I think, either in Yogyakarta or Aceh, health care workers still did not know much about hepatitis infection. They did not know the differences between hepatitis A, hepatitis B, and hepatitis C. Maybe they only know that hepatitis is a dangerous disease, but they do not realise how big the danger is because they have limited knowledge related to hepatitis' (Transcript 8).
	Most of the participants (72.3%) claimed that they received hepatitis B information (on infection and vaccination) from a health provider.		Ways to improve the skill of health providers	'Previously, we (vaccination programme of the Indonesian Ministry of Health) were quite helpful with the other programmes (Litbangkes) that conducted training with health care workers. This activity also involved a public figure who gave speech and training skills to health care workers about vaccination, including how to communicate effectively to the public' (Transcript 2).
Knowledge: These data represent the adult population`s knowledge concerning hepatitis B infection and hepatitis B vaccination	Mean of knowledge scores of hepatitis B infection and hepatitis B vaccination was 11 (95% CI: 10.65–11.37).	Knowledge of hepatitis B infection and vaccination	Population: knowledge of hepatitis B infection and vaccination among the population is low	'People's understanding of hepatitis is still low, so awareness is still lacking. This is because the current regulation on hepatitis B vaccination in Indonesia is only for infants and toddlers' (Transcript 11).
	The proportion of good knowledge about hepatitis B infection among participants was 19.5%, while 38.3% and 42.2% had fair and poor knowledge, respectively.			'First of all, they (the people) feel that vaccination is not important because they do not know the benefits of vaccination. Moreover, the cost of hepatitis B vaccination is expensive' (Transcript 11).

Only 19.3% of participants had good knowledge about adult hepatitis B vaccination. In addition, 41.5% and 39.2% of the participants had fair and poor knowledge of hepatitis B vaccination, respectively.		
344 of 757 (45.0%) unvaccinated participants claimed that one of the reasons for not being vaccinated was never hearing about the hepatitis B vaccination for adults before.	Ways to reinforce the knowledge	During the implementation (of child vaccination), we call it '*empep empep*'. This is a reminder for parents before the vaccination schedule is carried out by health workers (health promotion). We go around the village using motorbikes or cars to disseminate information to minimise missed opportunities. In this activity, we announced it through loudspeakers. We sometimes use loudspeakers from the mosque or village hall' (Transcript 5).
25.0% (n=187) of unvaccinated participants claimed that they never felt need hepatitis B vaccination.		'We (the health promotion staff) did an approach with parents (a father who rejects vaccination) and through communication that increases their knowledge about vaccination. We also involve *Aparatur Gampong* (community leaders in the village) to support vaccination programmes, such as providing additional meals during the vaccination schedule or providing a reward for a parent who brings their children for vaccination completely and punctually' (Transcript 5).

			Ways to reinforce the knowledge through cultural means	'There is a regular cultural event (*Rasulan*) in every village (in Gunungkidul) that provides *Wayang* (a traditional form of puppet theatre play that originated on the Indonesian island of Java) and several performing arts festivals. These shows are popular. Therefore, we plan to collaborate with this routine activity to provide health education through this event—for example, health education in *wayang* performances' (Transcript 7, page xx).
Reason to not vaccinate: Availability	21 (3.0%) unvaccinated participants claimed that one of the reasons for not being vaccinated was that the health facility that provides hepatitis B vaccination was too far from home.	Accessibility of hepatitis B vaccination	Platform: Geographic access to facilities	'There are areas that cannot be easily accessed, such as Gunung Batur and Lubuk Sari. Gunung Batur is an area that borders Klaten Regency. All of these areas are mountainous areas in Yogyakarta, which have limited geographical access (to health facilities that provide health facilities, including hepatitis B vaccination)' (Transcript 7).
			Governance: Regulation	'As far as I know, there is no regulation regarding hepatitis B vaccination for adults in Indonesia. The hepatitis B programme in Indonesia focuses on pregnant women first. We do not even know if there is a hepatitis B vaccine for adults or not. In addition, hepatitis B was not a trending topic compared to HIV' (Transcript 4).
			Do not know where to vaccinate.	'I think it (hepatitis B vaccination for adults) is necessary, especially for people like me (dentists). However, we do not know how to access the vaccination, nor do we know where to get the vaccine' (Transcript 4).

Insurance for hepatitis B vaccination	Only 17.8% (n = 159) of participants had insurance covering hepatitis B vaccination for adults.		Governance: Regulation	'Currently, Indonesia has two vaccination programmes, namely the national and elective programmes. Hepatitis B vaccination for adults is an optional programme. So, for adults who want to receive hepatitis B vaccination, they seek vaccination and pay for it themselves' (Transcript 2).
Willingness to pay for hepatitis B vaccination	640 participants (84.5%) claimed willingness to pay for hepatitis B vaccination.	Affordability of hepatitis B vaccination	Hepatitis B vaccination is expensive.	'In addition to low knowledge related to hepatitis B infection and vaccination, the cost of hepatitis B vaccination is also high (expensive). In my area (Takengon), I need Rp. 200,000 to Rp. 300,000 (14 USD to 21 USD) for each dose of hepatitis B vaccination (Transcript 3)'.
				'If the cost (hepatitis B vaccination for an adult) is charged to the public, there will be rejection. People are willing to pay to think that it directly impacts such a treatment. They feel better after paying for the treatment. However, they feel nothing after vaccination' (Transcript 7).
Reason to not vaccinate: cost of hepatitis B vaccination	There were 95 (13.0%) and 64 (8.0%) unvaccinated participants who claimed that one of the reasons for not being vaccinated was that there is no insurance to pay for the vaccine up take and hepatitis B vaccination fees are expensive.		Governance: Financing, insurance, and purchasing	'Most of the people in Gunung Kidul work as laborers (such as farmworkers) and do not have a fixed monthly income. So, they cannot afford hepatitis B vaccination' (Transcript 7).
Source of information related to hepatitis B infection and vaccination	The most common source of information related to hepatitis B infection and vaccination was health providers (n=527; 72.3%), followed by media (n=397; 54.2%) and family/relatives (n-161; 22.1%).	Dissemination of information	Tools: Equipment	'There are no tools (posters or banners) that talked about hepatitis B specifically. We published a banner about triple elimination (HIV-syphilis-hepatitis) in 2019, which targeted pregnant women only' (Transcript 4).

Type of media as source of information about hepatitis B infection and vaccination	The three most popular media sources that provide information about hepatitis B infection and vaccination were television (n=251; 54.2%), social media (n=215; 54.2%), and poster (n=88; 22.2%).		‘The most popular (type of dissemination) is social media. Before the pandemic, we (the health promotion programme) helped the hepatitis programme to disseminate information about hepatitis through exhibition billboards (owned by the Indonesian Ministry of Health) when celebrating Hepatitis Day’ (Transcript 1).
		Information systems: Lack of information	‘Honestly, our priority (hepatitis B program) right now only focuses on pregnant women, so the target programme in the regions (provinces) is limited to this population. Meanwhile, for the general population, we only disseminate information about hepatitis on World Hepatitis Day’ (Transcript 12).
		Way to improve dissemination: Tools	‘Previously, we only had leaflets (as tools for disseminating health information). If we (the health promotion programme) have enough money, we plan to develop more (leaflets) using Gayo (the local language of Takengon) because people do not understand medical language’ (Transcript 3).
			‘In Aceh, the most popular place is Warung Kopi (coffee shop). If we put up some information (banners or posters) about hepatitis B infection and vaccinations at the coffee shop, we believe many people could be exposed to that information’ (Transcript 8).

	Ways to improve dissemination: information system	‘Health promotion should be increased, which is not limited to hospitals or health facilities, but also in other places, such as mosques or radio. I have a regular schedule to talk about health at the mosque every month at *ba’da subuh* (after the evening prayer). In my opinion, this is one of the best ways to disseminate information, including health information’ (Transcript 3).
